# Editorial: Memory T Cells: Effectors, Regulators, and Implications for Transplant Tolerance

**DOI:** 10.3389/fimmu.2016.00007

**Published:** 2016-01-25

**Authors:** Yu-Qun Zeng, Chuanjian Lu, Zhenhua Dai

**Affiliations:** ^1^Section of Immunology, Second Affiliated Hospital of Guangzhou University of Chinese Medicine, Guangdong Provincial Academy of Chinese Medical Sciences, Guangzhou, China; ^2^Division of Dermatology, Second Affiliated Hospital of Guangzhou University of Chinese Medicine, Guangdong Provincial Academy of Chinese Medical Sciences, Guangzhou, China

**Keywords:** memory T cells, regulatory T cells, costimulation, migration, T cell metabolism, transplantation immunology

**The Editorial on the Research Topic**

**Memory T Cells: Effectors, Regulators, and Implications for Transplant Tolerance**

Memory T-cells respond to previously encountered antigens more rapidly and vigorously than their naive counterparts. They are divided into three subsets: central memory, effector memory, and tissue-resident memory T-cells. They are somewhat resistant to immunosuppressive treatments and are generally believed to be a threat to transplant survival. However, mounting evidence has demonstrated that memory CD8^+^CD122^+^ T-cells with central memory cell phenotypes (CD45RA^−^CD44^high^CD62L^high^CCR7^+^) can regulate T-cell homeostasis and suppress both autoimmune and alloimmune responses. Therefore, memory T-cells, especially CD8^+^CD122^+^ T-cells, may respond as either aggressive memory or regulatory T-cells (Treg). This research topic may shed light on when they act as memory versus Treg cells, and how to target memory T-cells or otherwise utilize memory-like Tregs to promote long-term allograft survival.

Memory T-cells are considered to be a major barrier to long-term transplant survival or tolerance (1). Targeting allospecific T-cell memory appears to be required for transplant tolerance induction. Then, the question is whether blocking conventional T-cell costimulation would inhibit memory T-cell responses. Previous studies have shown that memory T-cells are resistant to CD40/CD154 costimulatory blockade (2, 3). It is also generally accepted that B7-CD28 costimulation is not required for memory T-cell activation (4). They are either less dependent on or totally independent of CD28 costimulation (5, 6). Therefore, it is likely that blocking B7–CD28 is insufficient for preventing allograft rejection in the face of memory T-cells. Perhaps that is why a high incidence of acute rejection of renal allografts, despite CTLA4-Ig treatments, has occurred in clinic due to the cross-reactivity of memory T-cells, derived from pathogen-specific immune responses, with an alloantigen (7). However, recent studies using animal models have shown that optimal elaboration of secondary T-cell responses is dependent on B7–CD28 interactions in the context of anti-infectious immunity (Ville et al.). Interestingly, selectively targeting CD28 with FR104 is more potent in suppression of allograft rejection than targeting CD80/86 with CTLA4-Ig (Ville et al.), suggesting that selective blockade of CD28 signaling alone presents an advantage of allowing immunoregulatory signals mediated by CTLA4. Furthermore, blocking OX-40 costimulatory signal prolongs secondary heart allograft survival in the presence of CD40/CD40L and LFA-1/ICAM-1 blockade (8), indicating that additional blockade of OX-40 signaling is required for abrogating memory T cell responses.

Memory T-cells can rapidly trigger alloimmune responses (9). It has been known that early infiltration of CD8^+^ memory T-cells into allografts facilitates allograft rejection and presents a hurdle to achieving long-term allograft survival (10–12). Signaling pathways for memory T-cell migration to an inflamed graft include G protein-coupled chemokine receptor signaling and cognate antigen-engaged TCR signaling as both signals trigger downstream integrin activation (Zhang and Lakkis). Interestingly, cognate antigen presence is necessary for driving antigen-specific memory T-cell migration into the peripheral tissue even without acute inflammation (13). Blocking integrin with anti-LFA-1 or anti-VLA-4 mAb prevents memory T-cell migration to a graft, attenuates alloreactive memory T-cell recall responses, and suppresses allograft rejection (14, 15). However, indiscriminately blocking LFA-1, though preventing memory and effector T-cell migration, increases the chance of developing post-transplant EBV-associated lymphoproliferative diseases while targeting VLA-4 may result in reactivation of fatal infections (16). Therefore, it is important to seek new strategies, instead of the universal blockade of major chemokines, to prevent donor-specific memory T-cell migration without increasing the risk of infections. One potential strategy to do so is to target the inside-out signaling pathway downstream of the TCR but not chemokine receptors (Zhang and Lakkis), such as SKAP1, leading to the suppression of antigen-driven but not chemokine-driven memory T-cell migration to a graft.

Recently, there has been a renewed interest in immune metabolism in CD8^+^ T-cells. Their proliferation and function require a metabolic adaptation to meet their needs for energy and biosynthesis (Yap et al.). Activated CD8^+^ T-cells reprogram their metabolism from OXPHOS to aerobic glycolysis and glutaminolysis (17), supporting their rapid growth with sufficient energy as well as metabolic intermediates. Since glycolysis and glutaminolysis are two major metabolic pathways that are essential for CD8^+^ effector cell function, blocking metabolic pathways could lead to the discovery of new immunosuppressive drugs for preventing allograft rejection, although these approaches likely cause significant side effects. For instance, 2-Deoxy-d-glucose (2-DG) inhibits glycolysis by blocking hexokinase function and hence suppresses cytotoxic function of effector CD8 T-cells while blocking glutaminolysis with a glutamine antagonist 6-diazo-5-oxo-l-norleucine (DON) inhibits T-cell proliferation (Yap et al.). More studies are urgently needed to seek metabolic antagonists that are effective in immunosuppression but result in less severe side effects.

Previous studies have shown that CD8^+^CD122^+^ T-cells with central memory phenotypes regulate T-cell homeostasis (18) while more recent data have suggested that they suppress conventional T-cell responses (18–24) and control autoimmune diseases (25, 26). We have found that memory CD8^+^CD122^+^ T-cells and bystander central memory CD8^+^ T-cells inhibit murine allograft rejection (27, 28). Moreover, others have shown that central memory CD8^+^ T-cells mediate lung allograft acceptance (29). Importantly, we have demonstrated that memory-like CD8^+^CD122^+^ Tregs are more potent in their suppression of allograft rejection than their CD4^+^CD25^+^ counterparts (30). Hence, not only are memory CD8^+^CD122^+^ T-cells regulatory cells, they can also boost the strength of Treg-mediated suppression. The exact mechanisms underlying their suppression are still not well understood, although CD8^+^CD122^+^ Tregs may restrict immune responsiveness by production of IL-10, TGFβ1, and IFNγ. In order to utilize them in clinic transplantation, more extensive studies are required to fully understand their mechanisms of action and their safety. Furthermore, we have revealed that PD-1 expression on CD8^+^CD122^+^ T-cells is critical for their regulatory function. Therefore, memory-like CD8^+^CD122^+^PD-1^+^ T-cells could be one of the best Treg subsets for the induction of long-term allograft survival or tolerance. It remains to be determined whether this subset of Tregs can be expanded *in vitro*.

## Author Contributions

Y-QZ wrote primary draft, and CL and ZD edited the editorial.

## Conflict of Interest Statement

The authors declare that the research was conducted in the absence of any commercial or financial relationships that could be construed as a potential conflict of interest.
